# Imaging of Congestion in Cardio-renal Syndrome

**DOI:** 10.1007/s11897-025-00695-z

**Published:** 2025-02-25

**Authors:** Htet Htet Ei Khin, Joe J. Cuthbert, Abhilash Koratala, Giovanni Donato Aquaro, Nicola Riccardo Pugliese, Luna Gargani, Sokratis Stoumpos, John G. F. Cleland, Pierpaolo Pellicori

**Affiliations:** 1 School of Cardiovascular and Metabolic Health, University of Glasgow, Glasgow, UK; 2 Clinical Sciences Centre, Hull York Medical School, University of Hull, Cottingham Road, Kingston-Upon-Hull, East Yorkshire UK; 3 Division of Nephrology, Medical College of Wisconsin, Milwaukee, 53226 USA; 4 Academic Radiology Unit, Department of Surgical, Medical and Molecular Pathology and Critical Area, University of Pisa, Pisa, Italy; 5 Department of Clinical and Experimental Medicine, University of Pisa, Via Roma 67, Pisa, 56124 Italy; 6 Department of Surgical, Medical and Molecular Pathology and Critical Care Medicine, University of Pisa, Pisa, Italy; 7 Renal and Transplant Unit, Queen Elizabeth University Hospital, Glasgow, UK

**Keywords:** Congestion, Heart failure, Cardiorenal, Ultrasound, Jugular, Vena cava

## Abstract

**Purpose of Review:**

Both cardiac and renal dysfunction can lead to water overload - commonly referred to as “congestion”. Identification of congestion is difficult, especially when clinical signs are subtle.

**Recent Findings:**

As an extension of an echocardiographic examination, ultrasound can be used to identify intravascular (inferior vena cava diameter dilation, internal jugular vein distension or discontinuous venous renal flow) and tissue congestion (pulmonary B-lines). Combining assessment of cardiac structure, cardiac and renal function and measures of congestion informs the management of heart and kidney disease, which should improve patient outcomes.

**Summary:**

In this manuscript, we describe imaging techniques to identify and quantify congestion, clarify its origin, and potentially guide the management of patients with cardio-renal syndrome.

## Introduction

Cardiac and renal function are closely intertwined. Hypertension, diabetes and atherosclerosis, alone or in combination, are common causes of both heart and renal damage. Dysfunction of one organ will often affect the other, initiating a vicious cycle that leads to worsening cardiac and renal function [1]. This problem has even been given a name, the cardio-renal syndrome (CRS), of which there are at least five types [2]. 

Retention of water and salt, driven by neurohormonal activation in response to cardiac dysfunction leading to venous congestion is the hallmark of heart failure (HF), but may also occur due to renal dysfunction [3]. Congestion is responsible, at least in part, for symptoms and signs of heart failure, poor quality of life, worse outcomes, and substantial healthcare costs [4, 5]. Identification and quantification of congestion is challenging, especially when clinical signs are subtle. Even though congestion is common even in the early stages of chronic kidney disease (CKD) [6, 7], many patients do not receive specialist assessment until there is substantial or rapid deterioration in renal function. Sub-clinical congestion will often precede the onset of overt symptoms and is associated with a worse prognosis; timely detection of congestion may lead to earlier diagnosis and improved outcomes [8, 9]. Also, symptoms or signs of congestion, such as peripheral oedema or exertional breathlessness, are not specific to cardio-renal disease and are frequently attributed, rightly or wrongly, to ageing or comorbidities, such as obesity, varicose veins, or respiratory disease. Worse still, symptoms and signs may be treated with loop diuretics and not investigated further [10, 11]. 

In this article, we describe imaging techniques for non-invasive identification and quantification of congestion in patients with or at risk of developing CRS and suggest strategies to improve management.

## Chest X-ray

A chest X-ray (CxR) is a mandatory investigation in someone who presents with breathlessness. Although features such as cardiomegaly, valve or pericardial calcification, and pleural effusions are not diagnostic for heart failure, their presence indicates the need for more detailed cardiac investigations. In patients hospitalised with HF, features of pulmonary oedema and congestion are common and are associated with adverse outcomes [12]. However, the main function of a CxR in patients presenting with breathlessness is to identify respiratory conditions, such as pneumonia, pulmonary fibrosis or cancer, that may mimic heart failure.

## Echocardiography

Echocardiography is the most commonly used imaging tool for assessing cardiac structure and function. However, low levels of accuracy, the inter- and intra-observer variability, the need for expertise to perform a full and detailed echocardiogram, the complex and differing guideline recommendations for assessment and diagnosis of structural and functional cardiac dysfunction, and lengthy waiting lists contribute to delayed diagnosis in many patients with cardiorenal disease.

Some echocardiographic findings are common in patients with CKD and predict adverse clinical outcomes [13, 14]. For instance, the prevalence of left ventricular hypertrophy (LVH) increases with the severity of renal dysfunction, affecting up to 75% of those with an eGFR < 30 ml/min/1.73m^2^ or on dialysis [15, 16], and is associated with a greater risk of developing heart failure and death [17, 18]. 

Up to 50% of patients with CKD have heart failure. Assessment of left ventricular ejection fraction (LVEF) is important not only for risk stratification but also for treatment [19], although a normal LVEF in patients with CKD excludes neither impaired systolic function nor heart failure [16]. Global longitudinal strain (GLS), measured by two-dimensional (2D) speckle-tracking echocardiography (STE), may be a more sensitive index of myocardial systolic dysfunction than LVEF [20, 21]. Patients with CKD are likely to have reduced GLS [22, 23] that is associated with a lower eGFR [24], and a worse prognosis [25, 26], but there is no evidence that using GLS to guide the management improves outcomes.

Echocardiographic signs of diastolic dysfunction are also common in patients with CKD, particularly those with lower eGFR [27]. Left atrial (LA) dilatation is perhaps the most important (and most commonly overlooked) marker of diastolic dysfunction. The left atrium is a thin-walled structure in direct communication with the left ventricle and, as such, reflects the effects of both volume and pressure overload in patients with cardiac and renal disease. Of the many markers of diastolic dysfunction, LA dilatation has the strongest association with cardio-renal events in patients with cardiovascular risk factors and at all stages of CKD [28–32]. Sustained elevation in LA pressure will eventually cause pulmonary venous and arterial hypertension, followed by the development of right ventricular dysfunction and dilatation, and then right atrial hypertension leading to signs of systemic venous congestion [33]. Pulmonary and systemic venous congeston contribute to the onset and worsening of symptoms of heart failure, disease progression and, ultimately, death [34–36]. 

**Fig. 1:**
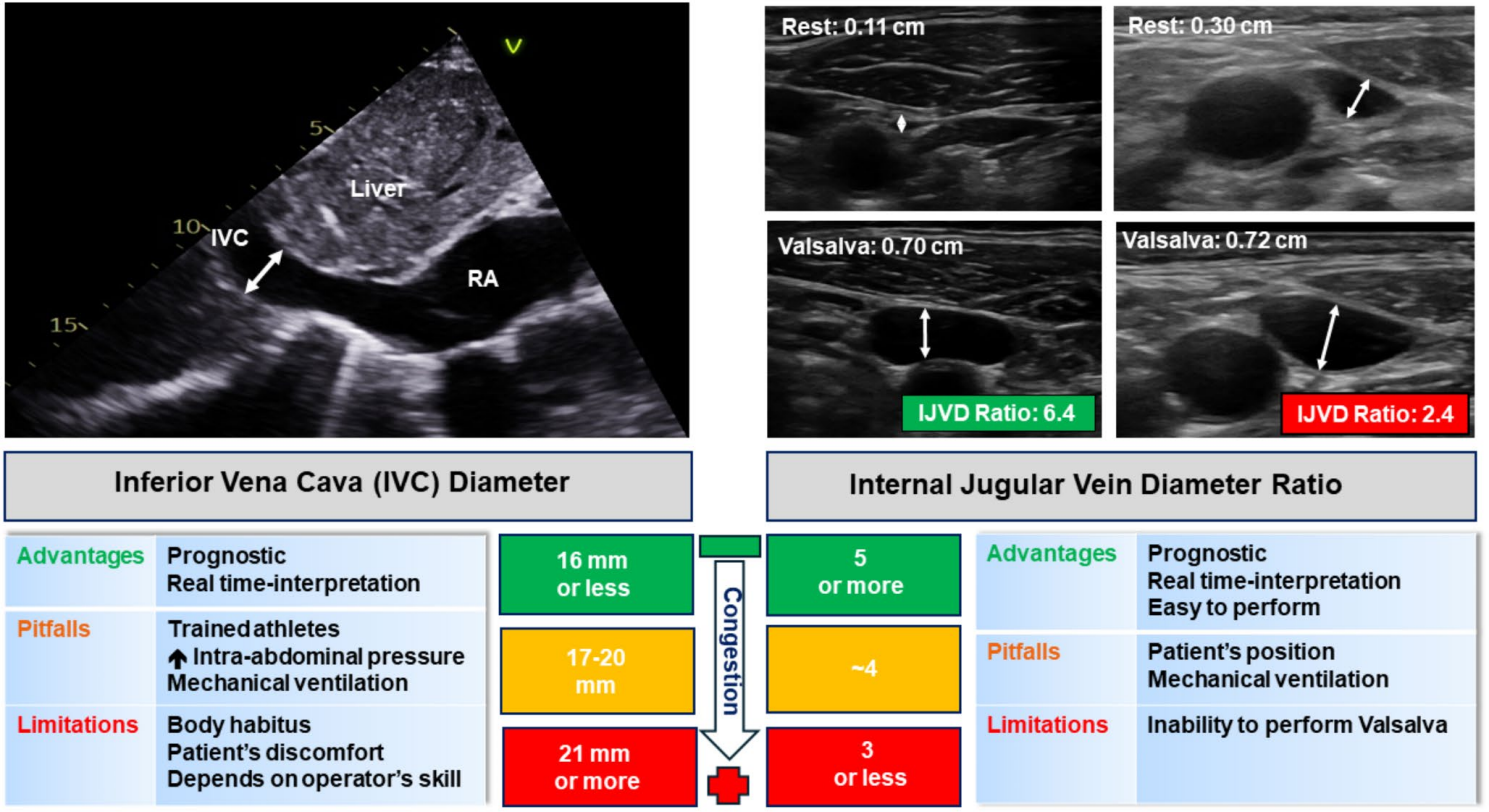
Advantages, pitfalls, and limitations of measuring inferior vena cava diameter (left panel) or internal jugular vein diameter ratio (IJVD Ratio, right panel). With worsening congestion, inferior vena cava diameter increases, and IJVD ratio decreases. Other abbreviations: RA– right atrium

## Inferior Vena Cava (IVC)

The inferior vena cava (IVC) is in continuity with the right atrium (RA) and can be visualised in most patients using ultrasound. Assessment of the size of IVC and the response to changes in intrathoracic pressure can be used to estimate RA pressure (RAP) [37]. An IVC diameter smaller than 2.1 cm, with a preserved (> 50%) inspiratory collapse, suggests normal RAP and absence of congestion (Fig. 1, *left panel*). In patients with heart failure, a dilated (> 2.1 cm) and stiff (< 50% inspiratory collapse) IVC is associated with worse symptoms and a higher risk of adverse cardio-renal outcomes [38–41]. Serial assessment of IVC dimension may guide fluid removal in patients on chronic renal dialysis and in those critically unwell receiving continuous renal replacement therapy [40, 42–44]. However, there are several limitations to using IVC ultrasound in isolation to identify and monitor congestion: technical skill and experience are required to achieve good image quality and interpretation of findings; and body habitus or low tolerability of abdominal pressure from the ultrasound probe might confound assessment of IVC [39, 45]. 

## Internal Jugular Vein (IJV)

The IJV is a superficial, large vein in the neck that can be easily scanned by ultrasound. The IJV should be assessed with the patient semi-recumbent with head and neck elevated at 45°. Compression of the IJV during examination should be avoided. At rest, at the end of the expiratory phase, IJV diameter is larger in patients who are congested but the difference can be quite subtle. During a Valsalva manoeuvre, IJV diameter is similar whether or not congestion is present [46]. Accordingly, a reduced ratio of IJV diameter during Valsalva compared to rest values (lower than 4, Fig. 1,* right panel*) is associated with more clinical evidence of congestion, a higher invasively-measured RAP and poorer outcomes in patients with chronic heart failure [37, 46–48]. Ultrasound assessment of the IJV may be easier than IVC assessment [46] and, as nephrologists routinely perform ultrasound assessment of neck veins when inserting dialysis catheters, there is the potential to integrate this technique into renal practice.

## Renal Venous Flow (RVF)

**Fig. 2:**
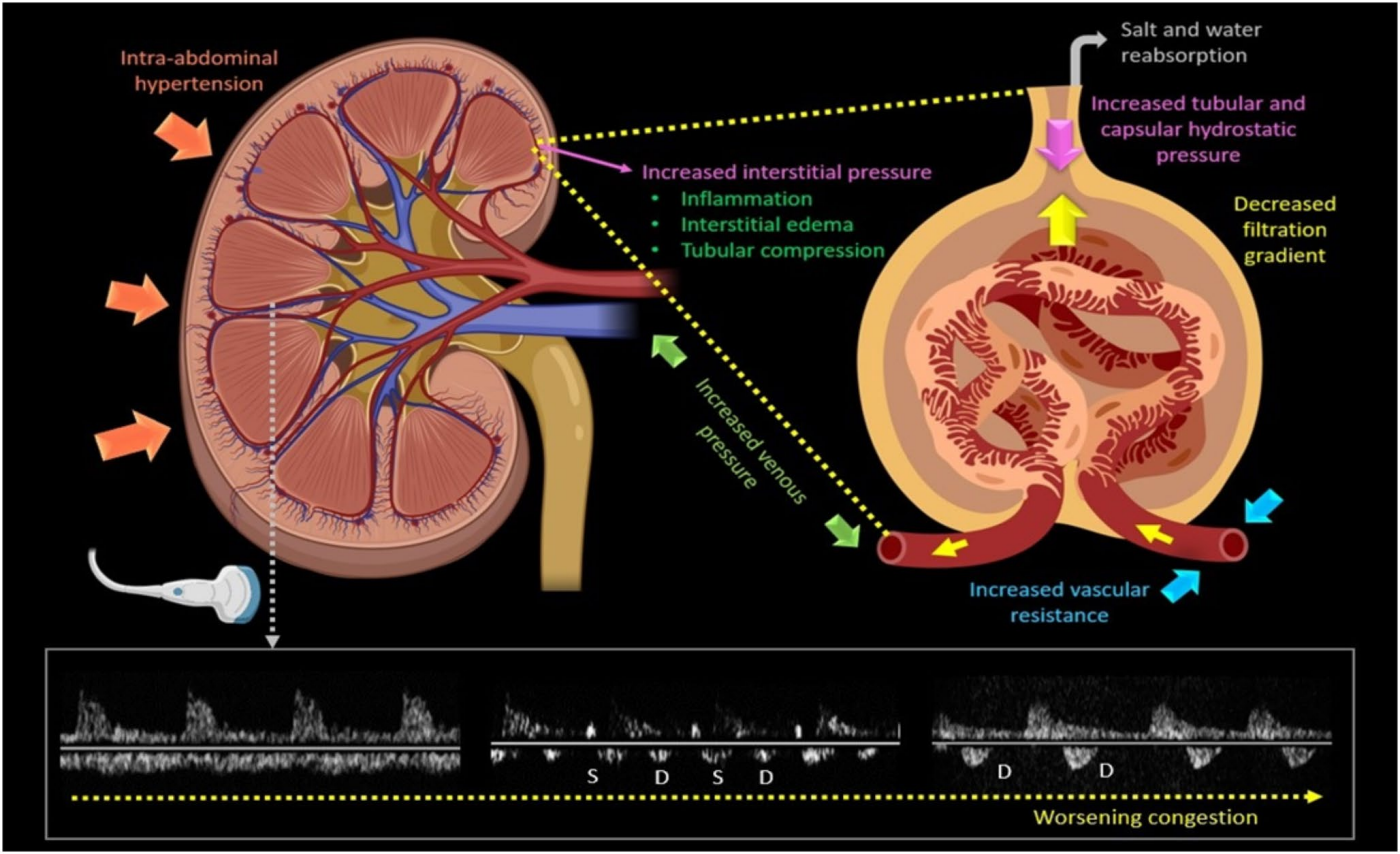
Pathophysiology of renal congestion and corresponding intrarenal Doppler patterns. As congestion worsens, renal venous flow (bottom, left to right) transitions from continuous to discontinuous. Initially, it becomes biphasic with distinct systolic (S) and diastolic (D) waves, and eventually, it becomes monophasic, with blood flow occurring only during diastole (D). *Figure made using Biorender®*

In patients with heart failure, it is the severity of venous congestion rather than cardiac dysfunction, that leads to worsening renal function [49–51]. Ultrasound can be used to assess both renal arterial flow and renal venous flow (RVF) [52], using pulsed-wave Doppler to sample flow within the interlobar veins during end-expiratory breath-hold [37]. In healthy individuals, RVF is continuous. As venous pressure increases, RVF becomes pulsatile with peaks during systole and diastole. As pressure increases further, RVF becomes monophasic, with blood flow only during diastole (Fig. 2). [53] In patients with HF, pulsatile and monophasic RVF is associated with a higher risk of HF admissions and CV death compared to those with continuous RVF [53, 54]. Interestingly, diuretic treatment can normalise RVF patterns in those who are congested due to cardiac dysfunction [55, 56]. The greater the proportion of the cardiac cycle with no RVF the worse the prognosis of HF [57–59].

## Hepatic Venous Waveform

**Fig. 3:**
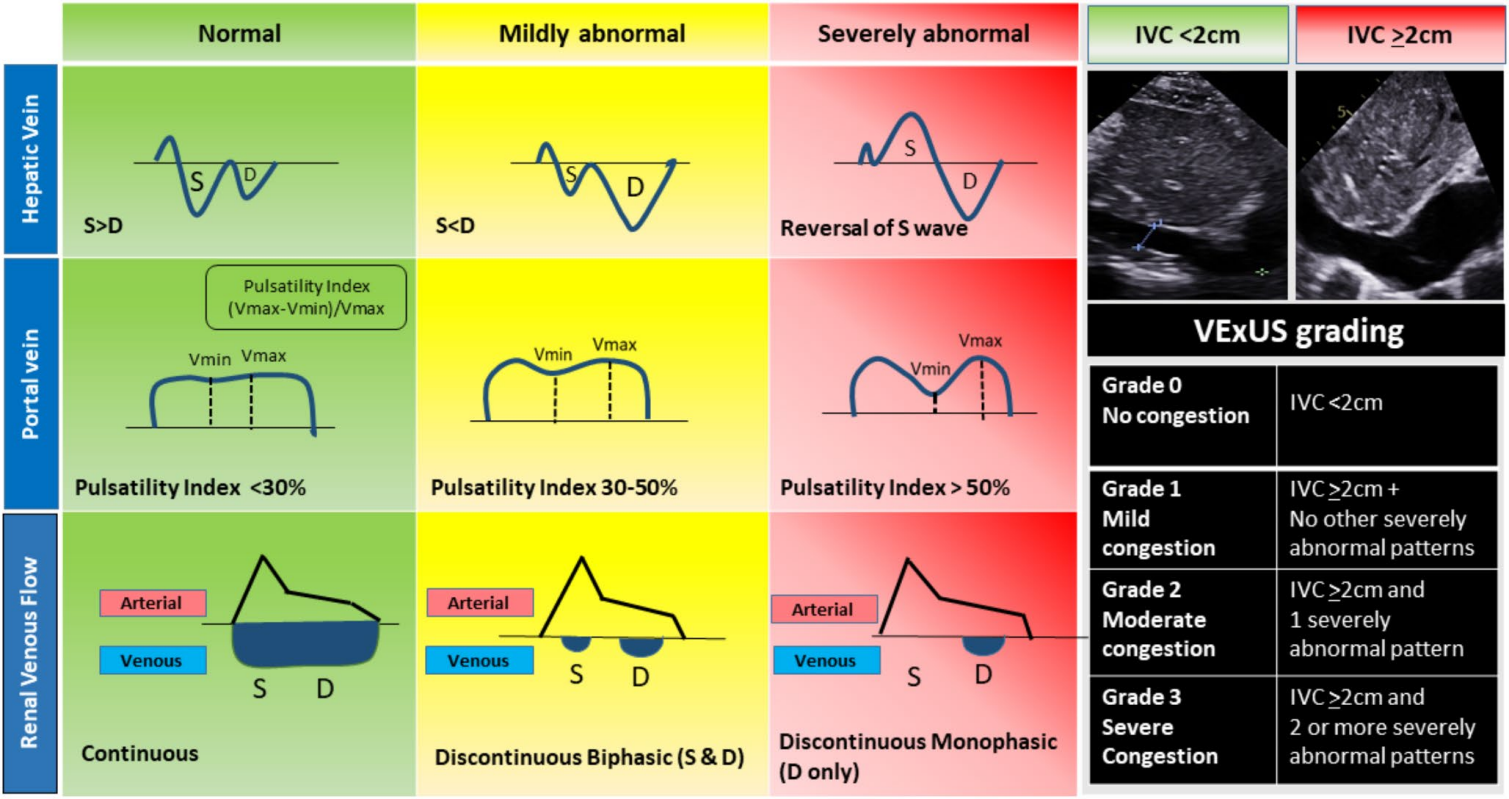
Components of the venous excess ultrasound grading system (VexUS) scoring system and grading. Abbreviations used: S– systole, D– diastole

Hepatic veins drain directly into the IVC and can be imaged through a subcostal window [60]. The hepatic venous waveform includes 4 waves named S (ventricular systole), V (transitional, atrial overfilling), D (ventricular diastole), and A (atrial systole). Typically, the S wave exhibits greater amplitude than the D wave (Fig. 3). With higher RAP, there is an inversion of S to D ratio (S < D pattern) or reversal of the S or V wave, which is associated with poorer outcomes in patients with cardiac or renal failure [61, 62].

## Portal Venous Waveform

The portal vein can be scanned through the same window as hepatic veins or IVC. Unlike hepatic veins, the portal vein normally has a continuous flow throughout the cardiac cycle. As RAP rises, the portal vein waveform becomes more pulsatile. A portal flow pulsatility fraction (defined as the difference between the maximal and the minimal velocities during the cardiac cycle, divided by the maximal velocity, Fig. 3) exceeding 30% is generally considered abnormal; a further increase to ≥ 50% is associated with right ventricular dysfunction, venous congestion, and risk of developing other clinical complications [63, 64]. 

## Venous Excess Ultrasound Grading System (VExUS)

The venous excess ultrasound grading system (VexUS) was developed to integrate multi-organ assessment of congestion by ultrasound; including evaluation of IVC diameter, hepatic, portal and renal venous flow [65]. When all findings are normal, the score is 0; a higher VexUS score is positively correlated with RAP (Fig. 3) [66]. While larger studies are required to determine the best way to integrate VexUS into the routine care of patients with CRS, recent data suggest that comprehensive monitoring of congestion by ultrasound might improve risk stratification and potentially guide management of patients with CRS [64, 67–69].

## Lung Ultrasound (LUS)

LUS detects extravascular fluid accumulation in the lungs, which appear as vertical echogenic lines extending from the pleura (B-lines), which is more sensitive than the presence of Kerley-B (septal) lines on a chest X ray [70–72]. LUS may facilitate diagnosis, risk stratification and management of congestion in patients with heart and renal failure [73]. A higher number of B-lines is associated with worse outcomes in patients undergoing haemodialysis [74–76] and in those with acute or chronic heart failure [77, 78]. In patients with heart failure, the number of B-lines decreases with treatment of congestion [79], and using LUS to guide treatment may reduce risk of worsening heart failure compared to standard care [80, 81]. LUS may also guide fluid removal in patients on renal dialysis [82]. 

However, B-lines are not specific to venous congestion: a high number of B-lines may be seen in patients with interstitial lung disease or non-cardiogenic pulmonary oedema [9, 83]. Moreover, the number of B-lines is lower in patients with heart failure who are obese [84]. Therefore, it is essential to integrate findings from LUS with other clinical, biochemical and imaging data before making clinical decisions.

## Magnetic Resonance Imaging (MRI)

Cardiac MRI is the gold standard method for assessing cardiac volumes and mass and for the non-invasive characterisation of myocardial tissue, including detection and quantification of interstitial oedema, scar and fibrosis [85, 86]. MRI can also be used to measure pulmonary blood volume, a surrogate for congestion [87]. Pulmonary transit time (PTT), the time that a bolus of intravenous contrast takes to pass from the RV into the LA, can be measured by MRI. PTT increases with higher pulmonary vascular resistance [88, 89], and can be used to estimate the pulmonary blood volume (PBV). Patients with a higher PBV indexed to body surface area (PBVi) have more severe pulmonary hypertension and congestion. In the PROVE-HF study that enrolled 112 outpatients with heart failure with a mean LVEF of 38%, those with a PBVi > 492 mL/m^2^ were at higher risk for adverse cardiovascular outcomes [90]. 

MRI can also be used to quantify interstitial water in extra-cardiac structures, including subcutaneous fat, skeletal muscle, the lymphatic system, kidneys, liver and spleen [85, 91] and to measure tissue perfusion [92]. Integrating these novel features of cardiac and extra-cardiac MRI into mechanistic clinical trials could provide valuable insights into the management of congestion with diuretics and other agents [93–95]. 

### The Value of an Integrated Exam– The Nephrologist’s View

Until recently, ensuring an adequate cardiac output and mean arterial pressure to maintain renal perfusion has been the focus of prevention and management of CRS. The key contribution of venous congestion to worsening renal function is now increasingly recognised. Although improvement in cardiac output after intensive medical therapy might have little acute effect on renal function [51], if cardiac function and congestion recover, renal function is also likely to improve [96, 97]. Ultimately, net renal perfusion pressure, the difference between renal arterial and venous pressures, rather than renal blood flow may be the more important determinant of renal function [98]. 

In a large cohort of outpatients with cardiovascular disease, a steep decline in eGFR was observed at central venous pressure (CVP) values higher than 6 mmHg, which was also associated with reduced survival [50]. There are many mechanisms by which venous congestion might affect renal function: elevated CVP is transmitted to the low-resistance renal venous system, leading to an increase in renal interstitial hydrostatic pressure. Interstitial oedema occurs when the renal lymphatic flow cannot keep up with the continued elevation of CVP. As kidneys are encapsulated organs, the kidney cannot swell to accommodate interstitial fluid, which results in a tamponade-like situation, compressing the glomeruli and tubules and reducing glomerular filtration [99–101]. Increased intra-abdominal pressure due to ascites or congestion of the splanchnic system may further exacerbate renal tamponade. The compensatory activation of renin-angiotensin-aldosterone system leading to sodium retention, endothelial dysfunction and renal arteriolar constriction adds to the problem. The term congestive nephropathy has been proposed to summarise these complex effects [102].

In addition to systemic venous congestion, elevation of left atrial pressures leading to increased extravascular lung water is also common in patients with advanced renal disease and associated with poorer outcomes [103, 104]. Furthermore, in disease states where clinical practice guidelines favour empiric administration of intravenous fluids, such as the hepatorenal syndrome, congestive organ injury is often overlooked. For instance, in a study including 127 patients with hepatorenal syndrome diagnosed by clinical criteria, 62% were found to have elevated right and left sided filling pressures on cardiac catheterisation. When these patients were switched from volume loading to diuretic therapy, renal function improved, suggesting that a diagnosis of congestive nephropathy had been missed [105]. 

Physical examination, assessment of water balance and, more controversially, changes in body weight are unreliable assessments of congestion. In a meta-analysis of 22 studies of patients presenting with breathlessness, the sensitivity for a diagnosis of HF for orthopnoea, peripheral oedema, jugular venous pressure, third heart sound, and lung crackles (rales) were only 50%, 51%, 39%, 13%, and 60%, respectively [106]. Documenting fluid balance is prone to errors in urine collection and water loss in breath, perspiration and faeces [107]. Provided weighing scales are accurate, the patient is mobile and there is strict attention to detail, measuring weight may be a useful method for monitoring congestion, although most of the excess water contributing to weight gain may be redistributed from the vascular space into the tissues. Changes in weight may reflect haemodynamic congestion poorly. Bioimpedance measurement of body composition may be more accurate than weight alone, although its unclear whether this reflects better scales, more care in taking measurements or the bioimpedance technology. Bioimpedance technologies is a simple and inexpensive technology with which to measure continuous cardiac output. It appears accurate, but its utility for assessing atrial pressures is less certain [98]. Biomarkers, such as natriuretic peptides, may also reflect congestion, but coexisting renal dysfunction may reduce their clearance, creating uncertainty about interpreting raised concentrations in the presence of advanced renal disease [108, 109]. Blood volume analysis using radio-isotopes is also feasible but the technology is not widely available and may be impractical for routine clinical practice.

With increasing evidence implicating the role of congestion in renal dysfunction, it is conceivable that objective assessment of congestion at the bedside using non-invasive methods could improve the diagnosis and management of patients with CRS. Point of care ultrasonography (POCUS) is increasingly used in patients undergoing renal dialysis [75, 110]. However, interpreting POCUS findings in isolation is prone to error. Therefore, a comprehensive approach known as ‘pump, pipes, and leaks’ has been proposed to evaluate the entire hemodynamic circuit rather than its individual components. It involves focused cardiac ultrasound (pump), including the stroke volume estimation, trans-mitral Doppler, mitral annular tissue Doppler; IVC and systemic venous Doppler [typically, hepatic, portal, and intrarenal veins] to estimate CVP and assess venous congestion (pipes), and assessment of extravascular lung water and ascites (leaks), which may be further supplemented by clinical assessment of peripheral oedema [111]. 

However, patient factors such as arrhythmias, obesity, liver disease, and an inability to breath-hold influence the acquisition and interpretation of many images. POCUS findings must be interpreted in clinical context, including medical history, symptoms and signs and laboratory data. Assessing intravascular volume and tissue water requires integration of information from several sources to formulate an individualised management plan.

### The Value of an Integrated Exam– The Cardiologist’s View

Identifying a patient with heart failure is difficult. Clinical symptoms and signs are part of the diagnosis but are frequently unrecognised or not investigated [10, 11, 112, 113]. Measurement of plasma natriuretic peptide concentrations is recommended by guidelines to help establish a diagnosis of HF [114], but many diseases that can mimic or exacerbate HF, such as anaemia, CKD, and atrial fibrillation, are also associated with raised NP concentrations [115]. These conditions are often associated with or cause HF, creating diagnostic uncertainty.

Classification of patients into phenotypes based on echocardiographic LVEF may be confounded by measurement errors, although application of artificial intelligence (AI) may reduce these [116]. There are many echocardiographic measures that are supposed to reflect diastolic dysfunction, but these are also prone to errors in measurement [117], and there is little consensus as to how they should be implemented in practice [118, 119]. Failure to consider HF as a diagnosis, lack of access to echocardiography and undue focus on ventricular function as opposed to atrial dilation may lead to long delays in the diagnosis of HF. Most patients with heart failure probably either die or deteriorate to the point of requiring hospital admission before a diagnosis is made [120]. A readily available, fast and accurate method of identifying patients with HF has been one of the greatest challenges facing some of the greatest cardiologists, including Sir Thomas Lewis (1881–1945) one of the scientific founders of cardiology.

The prevalence of HF is increasing, even though the diagnosis may be increasingly missed [121]. Despite improvements in medical and device therapies, mortality remains high. Venous congestion is a key driver of morbidity and mortality in patients with HF [122], and is the most common reason for hospital admission [123]. It is treatable [124, 125], and early intervention may improve outcomes [8, 126]. Defining HF by the presence of venous congestion is appealing but fraught [3], not least because venous congestion is notoriously difficult to identify on clinical grounds alone [127–130]. 

Ultrasound measures of congestion at the bedside have great potential for the diagnosis and management of patients with HF. However, no marker is without fault, and none can be used in isolation. Furthermore, systemic venous and pulmonary parenchymal congestion are manifestations of cardiac or renal dysfunction, the cause of which still needs to be ascertained. A distinction should be made between the severity of dysfunction of each organ and the severity of congestion as a consequence of organ dysfunction. Misdiagnosis and mis-management may be the consequence of focussing narrowly only on single organ dysfunction. The pathophysiology of venous congestion is complex and heterogeneous.

Integrated, comprehensive assessment is essential for accurate clinical interpretation of US measures of congestion, which requires considerable skill and expertise. However, with advent of AI to guide both the ultrasound examination itself and the interpretation of the results after integrating clinical information and blood tests, may democratise the use of POCUS. POCUS assessment is increasingly used in many specialties, including primary care. Practical education and instruction on the use of medical ultrasound should play a much larger role in under- and post-graduate medical education.

## Current Gaps and Future Directions

Ultrasound could transform patient care in cardio-renal medicine, especially in managing complex conditions like CRS. As evidence of its utility in CRS grows, large-scale, interdisciplinary studies involving cardiologists and nephrologists are required to establish diagnostic and therapeutic strategies that will support its broader adoption.

Implementation of ultrasound in routine renal clinical practice might not be straightforward. While cardiologists are well-versed in echocardiography, nephrologists lack a similar training. The growing evidence of the value of ultrasound for assessing congestion in CRS supports its inclusion in nephrology training programmes.

Advances in technology, particularly the integration of AI, could play a key role in overcoming skill gaps. AI-powered systems can assist clinicians in acquiring high-quality images, performing accurate measurements within seconds, and generating detailed reports. These tools could support structured training programmes, including remote education, accelerating skill acquisition even in resource-limited settings. The increasing availability of high quality, affordable handheld ultrasound probes that connect to smartphones or tablets could democratise access to ultrasound still further, bringing expert diagnostics to a much broader population.

In order to bridge these gaps, clinicians, researchers, industry and policymakers must align their efforts. Widespread acceptance of ultrasound in cardio-renal medicine will require a solid foundation of evidence, targeted training initiatives, and investment in affordable infrastructure.

## Conclusions

Congestion can be both a result and a cause of CRS; both congestion and CRS are associated with a high morbidity and mortality. Assessing congestion by clinical means alone is crude and imprecise. Detecting, quantifying and monitoring congestion by ultrasound, and perhaps other technologies, greatly improves accuracy. Organ imaging provides insights into the root causes of congestion. Ongoing research will clarify how best to integrate imaging with clinical and biochemical information, to optimise the management of patients with, or at risk of, CRS.

## Data Availability

No datasets were generated or analysed during the current study.
